# Lack of conspecific visual discrimination between second-year males and females in the Saffron Finch

**DOI:** 10.1371/journal.pone.0209549

**Published:** 2018-12-27

**Authors:** María Juliana Benítez Saldívar, Viviana Massoni

**Affiliations:** 1 Instituto de Ecología, Genética y Evolución de Buenos Aires, IEGEBA-CONICET, Buenos Aires, Argentina; 2 Departamento de Ecología, Genética y Evolución, Facultad de Cs. Exactas y Naturales, Universidad de Buenos Aires, Buenos Aires, Argentina; University of Sussex, UNITED KINGDOM

## Abstract

Sexually dichromatic birds often show delayed plumage maturation, but second-year (SY) males may or may not be distinguishable from females. In competitive contexts, SY males receive a reduced amount of adult males’ aggression, either by mimicking females or through signaling their sex and inexperience as subordinate males. To the human eye, reproductive dull SY male Saffron Finches are indistinguishable from females, whereas after second-year (ASY) males are golden yellow. Our aim is to establish whether SY males are sexually dichromatic with females to the eye of conspecifics. We describe plumage variation in females, SY and ASY males and, in particular, analyze assortative mating by color by comparing a previously disregarded yellow feather patch shared by the three groups. We measured plumage reflectance of the forehead, breast, belly, and axillaries, and used a two-step avian visual model analysis to estimate the ability of Saffron Finches to distinguish between SY males and females. We find that those groups are indistinguishable to conspecifics by color. Furthermore, we find non-significant evidence of assortative mating directly related to the coloration of comparable feather patches between females and each type of male, though body condition of SY males is associated to that of their mates. Our results are compatible with both the female-mimicry and the status signaling hypotheses of evolution and maintenance of delayed plumage maturation. However, the singing behavior of males reveals their presence within the breeding site; the combined effect of song and dull coloration suggest that SY males are honestly revealing their sex and status to conspecifics.

## Introduction

Birds are remarkably colorful [1,2]. A particularly striking feature of several species is their sexual dichromatism, i.e. the difference in color patterns between sexes. The extent of sexual dichromatism is frequently used as proxy of intensity of sexual selection acting on the species [3–5]. The conspicuous sex, usually males (but see [6,7]), would be under strong inter- or intrasexual selection, while the drabber sex would be selected for crypsis to reduce adult and nest depredation [8]. With the advent of reflectance spectrophotometry, objective measurements revealed that adults of several avian species formerly classified as monochromatic have cryptic sexual dichromatism, i.e., color differences between sexes undetectable by human vision [9–11]. Such is the case of several species within Thraupidae, the largest family of songbirds [8], which are now categorized as sexually dichromatic after the use of avian visual models [11].

Many species of songbirds show delayed plumage maturation (hereafter DPM), i.e. they acquire a definitive plumage color and pattern after their first potential breeding period [12]. In those species, adult secondary sexually dichromatic signals are reduced or absent in young individuals. Duller second-year-males (hereafter SY males) can reproduce successfully if sufficient resources are available [6,13,14], yet they remain at a competitive disadvantage with after second-year males (hereafter ASY males) over breeding resources or mates [15–17]. Two hypotheses, the female-mimicry hypothesis and the status-signaling hypothesis, have been suggested to explain delayed plumage maturation in birds in the context of social signaling. The female-mimicry hypothesis proposes that subadult males mimic the appearance of females, which in turn results in avoiding adult male-subadult male confrontations [18–20]. The status-signaling hypothesis assumes that subadult males are recognized both as males and subordinates by adults and, consequently, the former may receive fewer aggressions from the latter [12]. The competitive disadvantage of SY males in front of older males would arise as the result of females’ preference to mate with older and experienced males [17], from their subordinate status in front of older males [21], or both [21].

Assortative mating occurs when fitness depends on the similarity of mated pairs [22,23], when color traits are associated with timing of breeding or territory acquisition [24,25], or as the result of competitively superior males mating with females in better condition or larger size and lower quality males mating with the remaining less favored females [26]. Assortative mating in relation to body measurements, body condition, age, and visual signals has been reported in several bird species without (reviewed in [26–28]) and with DPM [21,29].

We investigated plumage coloration and body condition in the secondary cavity nester thraupid Saffron Finch, *Sicalis flaveola pelzelni* (P. L. Sclater 1872). Sexually mature SY males are indistinguishable from females to humans and remarkably different from ASY males in color and pattern. Females and SY males have a brown back with a pale whitish belly, while ASY males are overall golden yellow with an olive back streaked with black [30], unlike the subspecies *S*.*f*. *brasiliensis* in which mature individuals of both sexes are described as yellow plumaged and “dull” individuals as having only a yellowish ventral patch [31]. Females, and both type of males in *S*.*f*.*pelzelni*, in turn, have yellow axillaries (MJBS, this study). The yellow coloration is given by lutein [32], and is potentially under sexual selection pressure because carotenoid pigments are not produced endogenously but must be ingested [33]. Therefore, ASY males’ plumage and the yellow axillaries of the three groups could potentially serve a signaling function [34,35]. Saffron Finches’ ASY and SY males reproduce successfully in tree cavities, *Furnarius rufus* nests, and nest boxes [36–38]. In competitive contexts, plumage coloration in frontal body regions has been found to be important in social signaling [39–41] and differences in the forehead, breast, and belly could serve this purpose within and between sexes. Although all–and only–males sing a melodious and complex song [42], it still remains unknown whether the plumage color of Saffron Finches SY males and females is distinguishable to conspecifics. In addition, it is not known whether the coloration of yellow axillaries serves as a signal to mate assortatively by color or if it is related to body condition. If such is the case, we expect to find colorful females mated to ASY males and drab females mated to SY males.

Within this framework, we carried out spectrophotometric analyses in the Saffron Finch. We performed 1) a classic colorimetric analysis of plumage reflectance spectra of four body regions in females, SY and ASY males, 2) a perceptual analysis of colors using the tetrahedral color space, and 3) determined the conspecific ability to discriminate between females and both types of males using an avian visual system model coupled with a bootstrap procedure to link perceptual and statistical analysis. Finally, we explored the possibility of assortative mating based in the coloration of axillaries feathers and its relationship to body condition.

## Materials and methods

### Ethical statement

This study was carried out in strict accordance with the Guidelines for Ethical Research on Laboratory and Farm Animals and Wildlife Species and with the prior approval of the ethics committee of the Consejo Nacional de Investigaciones Científicas y Técnicas (CONICET, Resolution No. 1047 ANNEX II, 2005). All methods employed related to capturing, handling, and banding of the birds comply with the guidelines of the Dirección de Flora y Fauna (Buenos Aires province, Argentina). Specific permits to handle the birds were obtained from the Dirección de Flora y Fauna (permits No. 142/2013, 153/2014 and 65/2015). No animals were harmed during capture, and neither anesthesia nor euthanasia was required. The field studies did not involve endangered or protected species.

### Field methods

The Saffron Finch subspecies *S*. *f*. *pelzelni* is a small (*ca*.18 g), resident and granivorous passerine occurring from southern Brazil and eastern Bolivia to the south of La Pampa province in central Argentina [43].

Data from this study were collected during the breeding seasons (December-March) of 2013–2016, at an assemblage of 124 nest-boxes located in an agricultural landscape surrounding the Instituto de Investigaciones Biotecnológicas (IIB-INTECH), Buenos Aires Province, Argentina (35° 34′ S, 58° 01′W). The nest boxes were placed 1.3–1.7 m above the ground on fence posts delimiting paddocks, approximately at 30 m from each other. The paddocks are edged with tala (*Celtis tala*) and espinillo (*Acacia bonariensis*) native trees; the buildings are surrounded by a mixed forest of eucalyptus (*Eucalyptus* sp.), pine (*Pinus* sp.), honey locust (*Gledtisia triacanthos*), and tala.

We distinguished females from SY males as follows: only females incubate the eggs and have a brood patch while SY males have a cloacal protuberance and sing; ASY males were sexed by color, presence of cloacal protuberance, and singing behavior [38]. We captured females during incubation (*n* = 85) and ASY (*n* = 52) or SY (*n* = 40) mates when feeding nestlings inside the boxes, using a trap-system made with a swiveling piece of wood activated from a distance with a fishing line. The recapture of specimens born and banded in our study site (i.e., recruits) enabled us to identify drab males as SY males. In turn, SY males that return as yellow ASY males in the following season indicate that the immature plumage is worn for only one year [44].

### Color measurements

From each individual, we collected ~10 feathers from the forehead, breast, belly, and axillaries. Feathers from the first three regions were taken from the median line of the bird’s beak or head; feathers from axillaries were plucked from the only available point of extraction. Feather samples were collected into individual white paper envelopes and stored at room temperature and in the dark until mounting and spectrophotometric processing was performed in the laboratory. Feathers from each body region were stacked in the same orientation, with the distal side facing up, as seen on the bird, and mounted on matte black cardboard. An Ocean Optics USB 2000 spectrometer (Ocean Optics, Dunedin, FL, USA), sensitive to reflectance in the 300–700 nm range, with a bifurcated fiber optic probe (Ocean Optics, Dunedin, FL, U.S.A) and a PX-2 Xenon light source (Ocean Optics, Dunedin, FL, U.S.A.) were used to quantify feather coloration. The reflectance measurements were obtained relative to a white WS-1 Diffuse Reflectance Standard, recalibrated between measurements of each body region; the same matte black cardstock on which the feathers were mounted was used as the dark standard. OOIbase32 software was used to record and store the reflectance values; the probe was held at a 90° angle and 2 mm from the sample using a probe holder that blocked out all ambient light. Reflectance was measured at 3 randomly chosen points of each feather assemblage, lifting the probe between each measurement; the configuration parameters were integration time = 16 msec, scans to average = 50, and boxcar width = 10 [45,46]. The spectra were interpolated at 1 nm intervals over the range of 300–700 nm; we took the combined arithmetic average spectra for each body region of each individual [47,48].

After inspection of the reflectance curves (*e*.*g*., [49]) we measured informative and non-redundant variables for the type of plumage under study: brightness, hue, UV chroma and average chroma in the forehead; breast and belly of females and SY males; and brightness, UV chroma, carotenoid chroma, average chroma, and hue [50] in the yellow axillaries of the three groups and in ASY males’ forehead, breast and belly.

We considered brightness as the mean relative reflectance over the entire spectral range measured (300–700 nm), average chroma as the proportion of the difference between maximum and minimum reflectance/mean brightness [51], UV-chroma as the proportion of the total reflectance in the range 300–400 nm, carotenoid chroma as the proportion of the total reflectance in the range 450–700 nm [52,53] and hue as wavelength at the midpoint ([Rmax + Rmin]/2) in the reflectance spectrum [54,55].

We analyzed intrasexual differences in plumage coloration of Saffron Finch females, SY males and ASY males, and intersexual differences in plumage coloration of females and SY males with an avian visual model which incorporates sensitivity as cone absorbance [56,57]. All analyses were performed in the *pavo* package [58] in R version 3.4.1 [59], following the systematic procedure suggested by [60]. We applied visual modeling to consider the Blue Tit *Cyanistes caeruleus* visual system, i.e. an avian ultraviolet (UV) visual system, and set the models assuming illuminant conditions (D65) for open habitats [61], where the Saffron Finches forage and breed. We also quantified colors in the tetrahedral color space using spectral sensitivity functions measured in the Blue Tit [62]. Reflectance spectra are quantified in the tetrahedral color space [63] by estimating relative stimulation of the four types of cones that birds present. A reflectance spectrum is characterized by a vector, from which three variables or metrics can be defined. Two measurements (theta and phi) describe hue by calculating the angular location of a point in the tetrahedron, while r (saturation or chroma) refers to the length of the hue vector. We measured the intrasexual and intersexual chromatic (ΔS) and achromatic distances (ΔQ), assuming a Weber fraction of 0.1 for the long-wavelength sensitive photoreceptor [62], and relative cone proportions for the Blue Tit, wavelengths: UV = 1, short = 1.92, medium = 2.68, long = 2.70 [64]. Chromatic and achromatic contrasts are expressed in just noticeable differences (JNDs) and indicate how different are two spectra perceived given the visual space of the receiver; values > 1 are considered discernible by birds [56,65] only under extremely controlled conditions [66,67]. Therefore, we used a conservative value of > 2 JNDs as the threshold above which visual discrimination could take place [68,69].

### Statistical analyses

We used *t*-tests with Welch approximation for degrees of freedom, or Wilcoxon signed-rank test to compare color variables between SY males and females (Table 1). Axillaries’ color, the only patch that these groups share in coloration, was analyzed using one-way ANOVA and post hoc Tukey tests. In this patch, when assumptions for normality were not met, we performed Kruskal-Wallis test and calculated pairwise comparisons between groups. In addition, we compared color variables between females that paired with SY males and those that paired with ASY males. However, visual models are needed to quantify the subjective perception of color [56]. Therefore, the variability in coloration of each body region was evaluated by calculating the distances of all pairs of female-SY males and all pairs of individual distances within ASY males, females, and SY males groups (measured in JNDs). In order to determine if the axillaries of the three groups, and whether SY males’ and females’ body regions are both statistically and perceptually different to conspecifics, we conducted a two-step approach following [60]. First, we inspected, separately, the statistical differences for each body region by comparing the level of between-group variation relative to within-group variation with a distance-based PERMANOVA analysis. We considered this type of analysis as adequate because it accounts for the multivariate nature of data and, in addition, excludes the achromatic variation [60]. Initially, we checked for multivariate homogeneity of group dispersions (variances) using the ‘betadisper’ function. We obtained a null distribution to compare with our results, with 999 permutations; these tests were carried out with the function ‘adonis’ in the R-package ‘vegan’ [70]. Secondly, to analyze the perceptual difference between SY males and females, we used a bootstrap procedure to generate a distribution of mean distances by re-sampling the values of each group and body region and calculated the confidence interval for the observed distances [60]. We performed the same two-step analysis for the plumage patch of the axillaries. In order to quantify morphological variation, we measured tarsus length (± 0.1 mm) and mass (± 0.25 g) and used the residuals of the linear regression of mass and tarsus length as an index of body condition. We evaluated assortative mating by color traits and body condition in pairs formed by SY and ASY males using Pearson product-moment correlations. For each patch, *P*-values were corrected for multiple comparisons using Benjamini and Hochberg method for False Discovery Rate [71].

**Table 1:** Color description of Saffron Finches after second-year males, females, and second-year males, and comparisons between the latter two groups. Significant results are shown in bold.

	**ASY males**	**Females**	**SY males**	**Females *vs*. SY males**	
	(*n* = 52)	(*n* = 85)	(*n* = 40)	*t (*df*)	*P -value*
***Forehead***					
Brightness	15.03 ± 0.48	10.49 ± 0.24	10.85 ± 0.31	-0.92 (86.24)	0.67
Hue	519.36 ± 1.11	465.02 ± 8.93	474.13 ± 5.52	1862	0.67
UV Chroma	0.13 ± 0.01	0.12 ± 0.01	0.12 ± 0.01	-0.54 (86.54)	0.67
Average Chroma		1.41 ± 0.03	1.44 ± 0.05	-0.43 (69.75)	0.67
Carotenoid Chroma	-0.76 ± 0.01				
***Breast***					
Brightness	22.35 ± 0.68	25.09 ± 0.65	24.95 ± 1.00	0.12 (72.35)	0.99
Hue	502.02 ± 5.07	383.00 ± 4.00	380.27 ± 5.16	1825	0.99
UV Chroma	0.15 ± 0.01	0.15 ± 0.01	0.14 ± 0.01	-0.01 (86.57)	0.99
Average Chroma		1.18 ± 0.02	1.22 ± 0.03	-1.09 (85.64)	0.99
Carotenoid Chroma	-0.81 ± 0.01				
***Belly***					
Brightness	26.47 ± 0.72	32.06 ± 0.70	32.34 ± 1.00	-0.22(77.41)	0.82
Hue	464.6 ± 9.6	349.92 ± 1.53	349.97 ± 1.95	1647	0.82
UV Chroma	0.17 ± 0.01	0.17 ± 0.01	0.16 ± 0.01	1.71(75.68)	0.18
Average Chroma		1.05 ± 0.02	1.12 ± 0.03	1.61(0.11)	0.16
Carotenoid Chroma	-0.74 ± 0.01				
	**ASY males**	**Females**	**SY males**		
	(*n* = 52)	(*n* = 85)	(*n* = 40)	*F(df)*	*P-value*
***Axillaries***					
Brightness	26.37 ± 0.98^a^	24.98 ± 0.60^b^	23.12 ± 0.90^b^	3.23(2,174)	**0.04**
Hue	406.02 ±10.17^a^	425.05 ± 11.49^a^	408.13 ± 7.05^a^	1.81(2)	0.4
UV Chroma	0.20 ± 0.01^a^	0.19 ± 0.01^b^	0.18 ± 0.01^b^	5.11 (2,153)	**0.007**
Average Chroma	1.12 ± 0.04^a^	1.01 ± 0.03^b^	1.14 ± 0.04^a^	5.81(2,172)	**0.003**
Carotenoid Chroma	-0.65 ± 0.01^b^	-0.43 ± 0.01^a^	-0.48 ± 0.02^a^	62.02(2,174)	**<0.001**

* Welch t-test statistic (*df* = degrees of freedom). *P*-values were corrected for multiple comparisons. Different letters (^a^ and^b^) indicate significant difference between groups.

## Results

### Reflectance spectra and classic colorimetric variables

**Fig 1:**
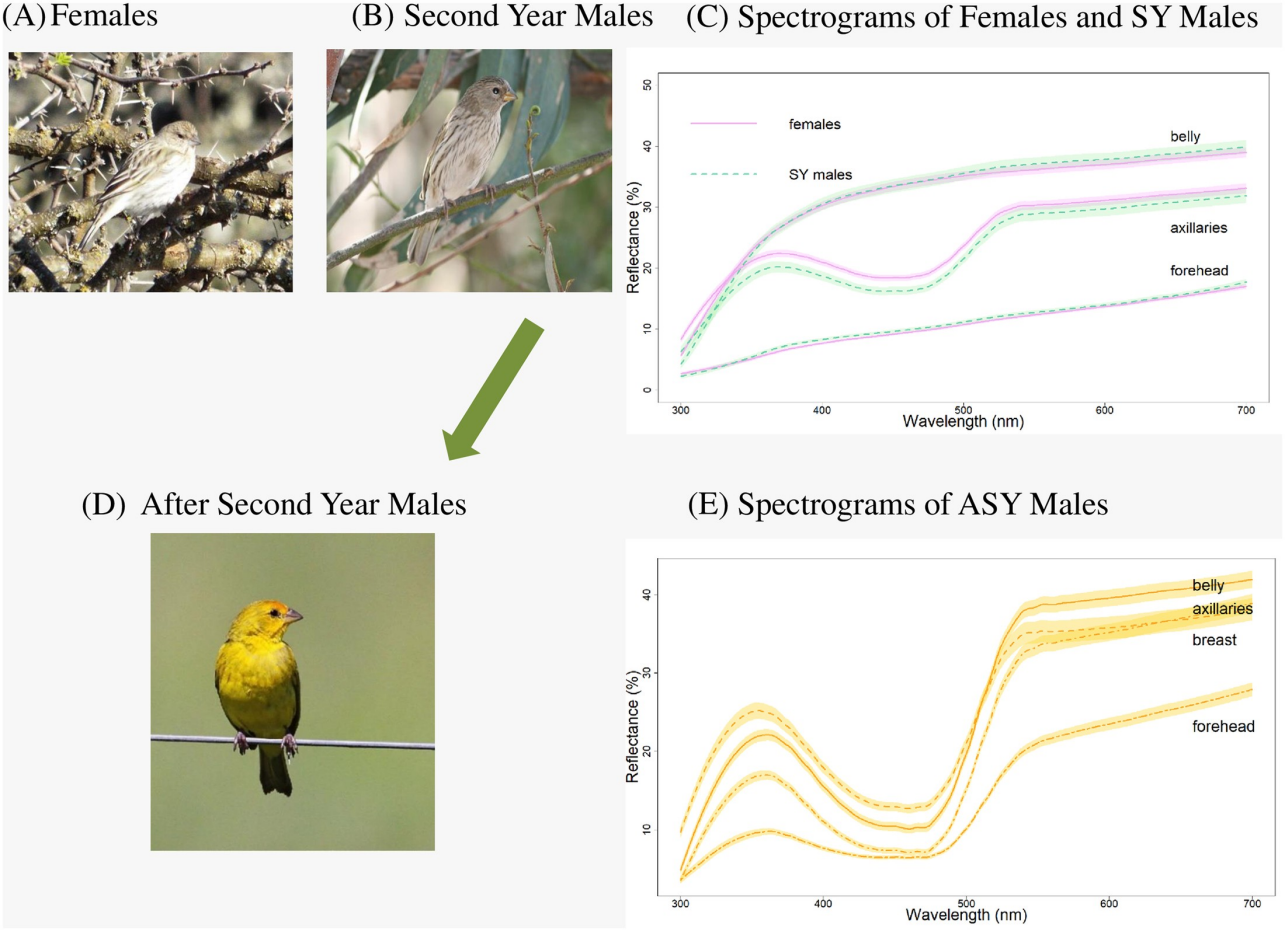
Saffron Finch females and second-year males are indistinguishable to humans and to statistical and perceptual tests (Fig 1A and 1B) but different from after second-year males (Fig 1D). **Fig 1C shows mean reflectance spectra (± *s*.*e*.) of females (*n* = 85) and second-year males (*n* = 40), and Fig 1E shows the mean reflectance spectra (± *s*.*e*.) of after second-year males (*n* = 52)**. Spectrogram of females and SY males’ breast was omitted to avoid line crowding with those of the belly.

Second-year male and female spectra overlapped in the belly, breast, and forehead, but female axillaries showed higher reflectance than those of SY males (Table 1). Saffron Finch ASY males showed the reflectance spectra expected for carotenoid pigmentation in all the regions measured; the reflectance of the UV peak was particularly variable among regions, with the lowest and highest UV peak in the forehead and the axillaries, respectively (Fig 1D and 1E).

There were no significant differences between females and SY males in belly, breast and forehead in the studied variables (Table 1). In the axillaries, the analysis of variances showed significant differences in most variables, including UV chroma (*F*_2,153_ = 5.11, *P* = 0.007), carotenoid chroma (*F*_2,174_ = 62.02, *P* = 0.004), average chroma (*F*_2,172_ = 5.81, *P* = 0.004) and brightness (*F*_2,174_ = 3.23, *P* = 0.04).

### Tetrahedral color space analyses

**Fig 2:**
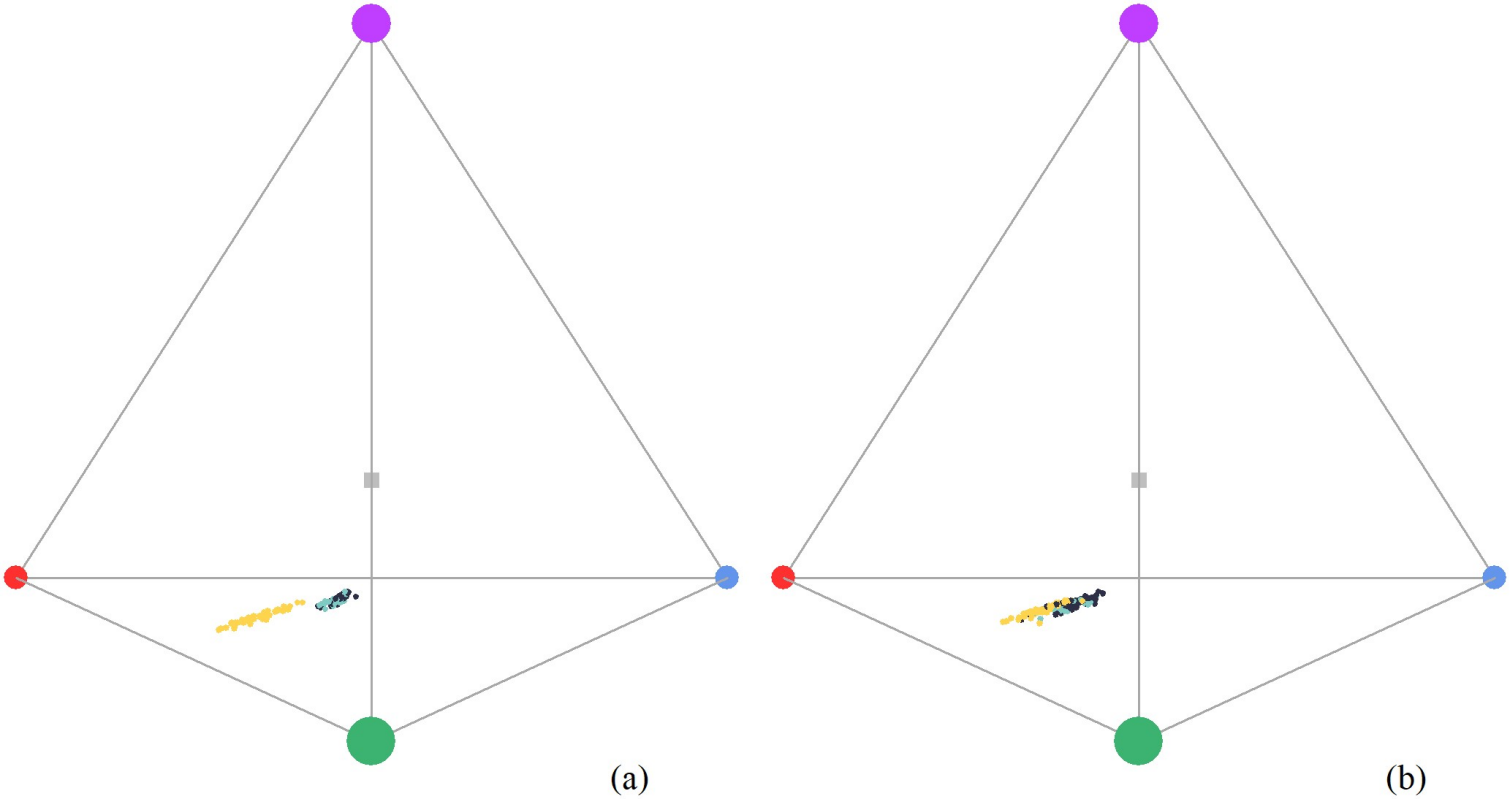
(A). Belly plumage color spectra in the tetrahedral color space. Each circle represents an individual. Vertices of the tetrahedron indicate type of cone: Ultraviolet (violet), short (blue), medium (green), long (red). Yellow circles are after second-year males, blue circles are females, teal circles are second-year males, and the grey square represents the achromatic origin or center of the tetrahedron. (B). Axillaries plumage color spectra in the tetrahedral color space. Each circle represents an individual. Vertices of the tetrahedron indicate type of cone.

The analyses of the perceptual characteristics of the colors of the three groups showed that the hue vectors (hue longitude θ, and UV- hue latitude ϕ) for all the body regions of SY males, females, and ASY males were located toward the u-m-l (ultraviolet-medium-long wavelength) region of the tetrahedron. The data points of SY males and females were always clumped together in a cluster in the tetrahedron, and closer to the achromatic origin of it, which indicates the chroma of feathers hues is relatively low (Fig 2A). The light reflected by SY males, females and ASY males stimulated medium and long wavelength cones the most; both yellow and dull birds showed variance along the UV- hue latitude component (Fig 2B).

#### Visual models comparisons within and between sexes

For each body region we made 3570 among female comparisons (*n* = 85 females), 780 among-SY males comparisons (*n* = 40), 1326 among-ASY males comparisons (*n* = 52), and 3400 comparisons between SY males-females.

Visual model analyses showed that ASY males have above threshold variability in the chromatic components in all regions studied (all values > 2 JNDs, Table 2). Females and SY males, however, showed relatively low chromatic distances in the four regions analyzed (all values < 2 JNDs), except in the forehead of females. All achromatic contrasts have values 2 > JNDs; in particular, ASY males’ axillaries had the highest mean value.

**Table 2:** Chromatic and achromatic distances of reflectance spectra among after second-year males, females and second-year males, and between second-year males and females. Mean ± *s*.*e*. of just noticeable differences (JNDs).

		ΔS				ΔQ		
	ASY males	Females	SY males	SY males *vs*. Females	ASY males	Females	SY males	SY males vs. Females
*Forehead*	2.35 ± 0.04	2.57 ± 0.04	1.40 ± 0.03	1.63 ±0.02	2.43 ± 0.05	2.24 ± 0.03	2.10 ± 0.06	2.17 ± 0.03
*Axillaries*	2.23 ± 0.04	1.88 ± 0.02	1.70 ± 0.04	1.74 ± 0.02	3.28 ± 0.07	2.58 ± 0.03	2.70 ± 0.07	2.67 ± 0.03
*Breast*	2.46 ± 0.05	1.63 ± 0.02	1.42 ± 0.05	1.36 ± 0.02	2.71 ± 0.05	2.82 ± 0.04	2.83 ± 0.07	2.82 ± 0.03
*Belly*	2.73 ± 0.05	1.03 ± 0.01	0.87 ± 0.02	0.85 ± 0.01	2.22 ± 0.05	2.31 ± 0.03	2.12 ± 0.05	2.20 ± 0.03

When comparing SY males and females, the results of the betadisper function showed homogeneous dispersion between these two groups (Forehead: *F*_1,123_ = 1.41, *P* = 0.24, Breast: *F*_1,123_ = 0.24, *P* = 0.62, Belly: *F*_1,123_ = 0.02, *P* = 0.86, Axillaries: *F*_1,123_ = 0.11, *P* = 0.74), which allowed us to continue with the PERMANOVA analysis; the latter showed significant statistical differences between groups only in the chromatic component of axillaries (PERMANOVA: *F*_1, 123_ = 3.34, *P* < 0.04), but not in the forehead (*F*_1,123_ = 2.66, *P* = 0.09), breast (*F*_1,123_ = 2.89, *P* = 0.07) or belly (*F*_1,123_ = 2.24, *P* = 0.13). Subsequent perceptual bootstraps of group separation based on the axillaries color showed a mean ΔS of 0.54 with 95% confidence intervals (CI) of 0.19–1.01, that fell below the discrimination threshold of 2 JNDs. The comparison of axillaries between ASY males and females could not be computed since the betadisper function showed heterogeneous variance dispersion between these two groups (*F*_1,135_ = 5.78, *P* = 0.02). Finally, the comparison of the axillaries between SY males and ASY males showed homogeneous dispersion of these groups in this patch (*F*_1,90_ = 3.34, *P* = 0.07) and statistical differences in the PERMANOVA analysis (*F*_1,90_ = 48.48, *P* < 0.001), with resultant bootstrap analysis showed a mean of ΔS = 2.50 with CI (1.88–3.14).

### Assortative mating

We found significant correlations (after controlling for multiple comparisons) between females mated with SY males in the hue of foreheads and breasts (Table 3). However, we found no significant proof of assortative mating between females and ASY mates regarding the axillaries plumage. Females that pair with ASY males had higher UV chroma than those that pair with SY males in axillaries but, after correcting the *P*-value for false discovery rate, the result was non-significant.

**Table 3:** Correlations of color variables in pairs formed by after second-year and second-year males. Pearson product-moment correlations; significant results are shown in bold. *P*-values were corrected for multiple comparisons using Benjamini and Hochberg method for False Discovery Rate.

	ASY males and females		SY males and females	
	(*n* = 41 pairs)		(*n* = 29 pairs)	
	*P*-value	*r*	*P*-value	*r*
***Forehead***				
Brightness			0.09	0.34
Average Chroma			0.08	0.37
UV Chroma			0.08	0.37
Carotenoid Chroma			0.3	0.2
Hue			**1.115E-05**	0.75
***Axillaries***				
Brightness	0.47	-0.12	0.24	0.3
Average Chroma	0.25	0.3	0.24	0.24
UV Chroma	0.375	0.23	0.15	0.41
Carotenoid Chroma	0.47	-0.13	0.24	-0.23
Hue	0.47	0.15	0.24	0.27
***Breast***				
Brightness			0.27	-0.27
Average Chroma			0.23	0.32
UV Chroma			0.69	0.11
Carotenoid Chroma			0.81	-0.05
Hue			**0.02**	0.52
***Belly***				
Brightness			0.95	-0.02
Average Chroma			0.95	-0.05
UV Chroma			0.95	0.01
Carotenoid Chroma			0.35	0.34
Hue			0.45	0.26

The only plumage patch shared between females and SY males are the axillaries; other comparisons would be meaningless.

**Fig 3:**
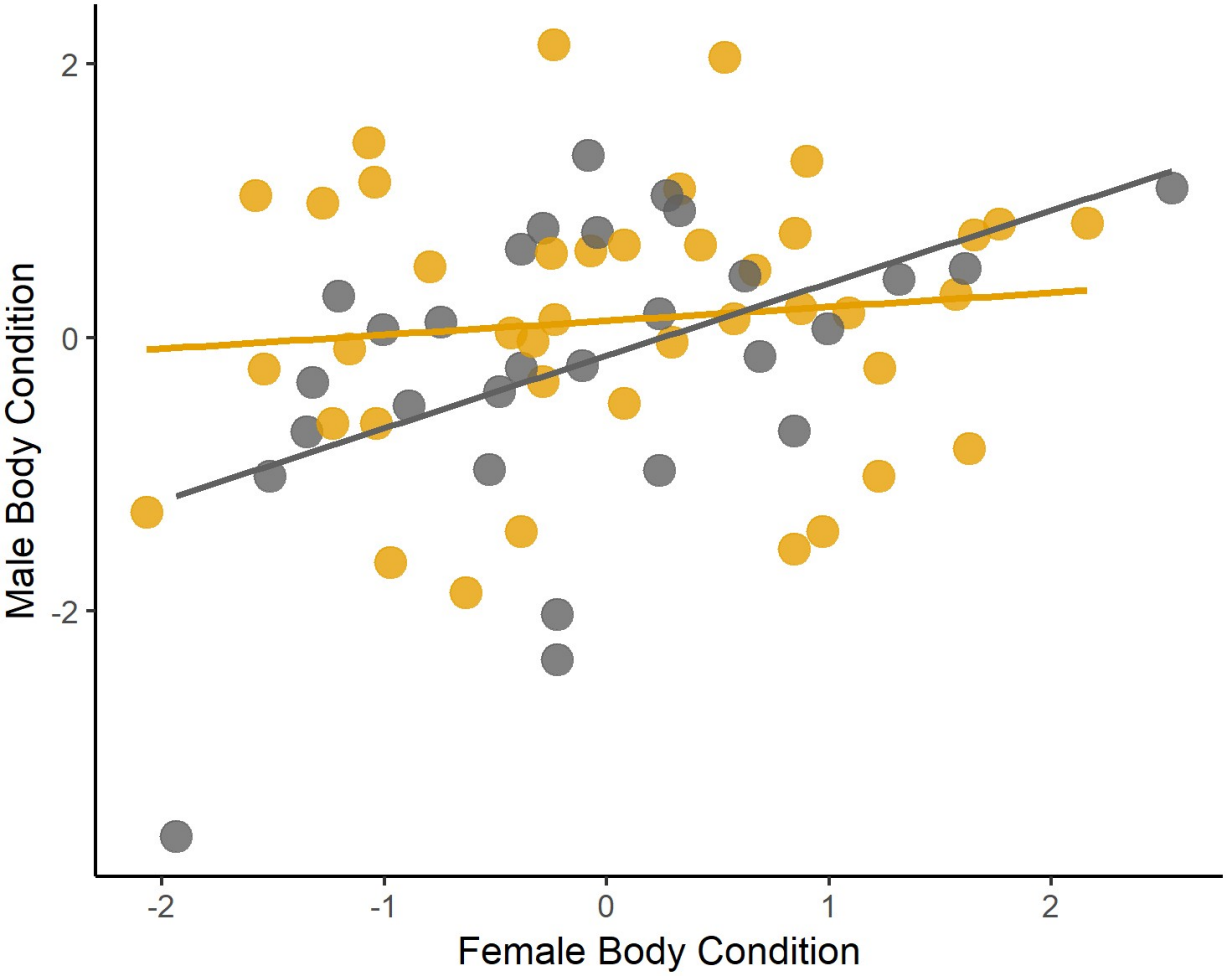
Relationship between female and male body condition within pairs split into 2 classes. After second-year males’ pairs (yellow circles) (*R*^2^ = 0.11, *P* = 0.49, *n* = 41) and second-year males’ pairs (grey circles) (*R*^2^ = 0.48, *P* = 0.008, *n* = 29).

Saffron Finches did pair assortatively by body condition (*R*^2^ = 0.28, *P* = 0.02, *n* = 70). However, when splitting the data by male plumage coloration, we realized that the significant association was driven by a positive relationship between females and SY males body condition (*R*^2^ = 0.48, *P* = 0.008, *n* = 29), and there was no association between the body condition of females and their ASY mates (*R*^2^ = 0.11, *P* = 0.49, *n* = 41) (Fig 3).

## Discussion

In the present study, we described plumage coloration in the Saffron Finch, a dichromatic secondary cavity nester with DPM. We analyzed the plumage of females, SY and ASY males and evaluated assortative mating by color and body condition. We found that females and SY males are indistinguishable to conspecifics by color and weak or otherwise non-relevant signals of assortative mating by color between ASY and SY males’ pairs. Second-year males and females did pair assortatively by body condition.

We found above threshold distinguishability in the chromatic components of all body regions in ASY males (Fig 1E). These differences may convey information about the quality of the signalers [72], suggesting that their yellow coloration (given by lutein acquired through diet) is probably the subject to inter- or intrasexual selection [73,74].

Several studies have found that female coloration is important in male mate choice [75,76], reproductive success ([77–79], but see [80]), and parental care [79]. Females of several dichromatic species display ornamentation that is similar to males, but often reduced in intensity or extent [32,34]. The finding that both females and SY males wear a previously disregarded yellow feather patch (MJBS, this work) raised the question of whether that trait served a signaling function [34]. If dull body regions do not show any differences between SY males and females, and if, as assumed by us, their axillaries convey useful information for courtship, that feather patch could allow silent SY males to go undetected by ASY males and still enable mutual mate choice during courtship.

The tristimulus color analysis showed typical carotenoid reflectance curves for ASY males and curves with no clear peak for all but one body region of SY males and females, the axillaries. Regular statistical comparisons found significant differences between the latter two groups in the hue of the forehead and breast. In addition, significant differences among and between groups in chroma and brightness of axillaries were found, suggesting this area had the potential to be a conspecific visual signal. However, the tristimulus “hue” may not correspond to the “hue” actually perceived by the receiver [81]. Thus, we proceeded with the tetrahedral color space analysis. The latter showed no separation in the color space location between SY males’ and females’ data points at any body region, including the axillaries. All spectra were close to the achromatic center, showing relatively low chroma. In addition, all color data points were located between the red-green cone wavelengths and spread along a vertical line toward the UV-cone vertex, but failed to separate SY males from females’ data points. The use of an avian visual model [56] coupled with a two-step analysis to assess statistically and perceptually significant differences among these groups [60] showed that, a) the homogeneity of variances within and between groups only allowed meaningful comparisons for the chromatic component of axillaries between ASY and SY males, and between SY males and females, and b) bootstraps of group separation showed perceptual intrasexual differences in axillaries coloration of ASY and SY males, and below threshold intersexual differences between SY males and females, suggesting the latter two groups are likely indistinguishable to conspecifics by color.

Saffron Finches breeding in Chascomús and other southern latitudes differ from the Brazilian subspecies, *S*.*f*, *brasiliensis*. In the latter, young individuals of both sexes show DPM, turn into brighter yellow plumage before the second (males) or following reproductive seasons (some females), and mate assortatively by color according to a tristimulus color analysis based on photography [31]. In the southern subspecies, however, only males show DPM and, along with females, are highly dichromatic with adult yellow plumaged males. The tristimulus and perceptual discrimination color analyses based on reflectance spectrophotometry failed to discriminate between females and SY males in *S*.*f*. *pelzelni* (this study). Our results are consistent with the finding of a high degree of overlap between morphs of unornamented male and female Red-backed Fairywrens *Malurus melanocephalus* [82], but differ with the sexual dichromatism found in the Tawny-bellied seedeater *Sporophila hypoxantha* DPM plumage [83]. The 1 JND threshold used by the authors, together with differences in the statistical analyses employed, however, suggest the latter may not have sexually dichromatic plumage during the second year of life. Unlike other subtropical birds with delayed plumage maturation (Tawny-bellied seedeater, [83], Long-tailed manakins *Chiroxiphia linearis* [84]), but akin to White-bearded manakins *Manacus manacus* [85], our banding records reveal that ASY males plumage is acquired after only one potential breeding season [37,86], as occurs in the males of the Brazilian subspecies. Contrary to what happens with the House Finch *Carpodacus mexicanus* [87], early reproduction as SY males in Saffron Finches does not prevent them from molting into the definitive ASY plumage [44]. Unfortunately, we were unable to compare the color of ASY males that reproduced as SY males to that of ASY males that did not. Evaluating if early reproduction is related to subsequent plumage color production [88] or survival [89] should be the aim of future studies.

The secondary cavity nester habit coupled with sexual dichromatism and DPM suggest both sexes in the Saffron Finch may experience intense intrasexual competition to breed. When limiting resources become available, adult-plumaged males of species with DPM usually obtain higher quality territories and mates than young, drab males [20,90], and DPM males would be at competitive disadvantage in front of ASY males (reviewed in [20]). Therefore, we expected to find colorful females mated to ASY males and drab females mated to SY males. Our results did not support that prediction; females paired with SY vs. ASY males were indistinguishable in color, and ASY and SY males’ mates were not associated by the color of axillaries. We found positive correlations between SY males and their mates in the hue of foreheads and breasts; however, these results should be taken with caution because the statistical and perceptual analyses suggest Saffron Finches are unable to discriminate between these groups in terms of plumage coloration. We interpret our results as providing weak to null support of assortative mating directly related to body coloration in this subspecies.

Body condition represents the size of an individual’s energy reserves relative to its body size [91], and is considered as an indicator of quality because it may affect the individual’s survival ([92] but see [93]) and reproduction in several bird species [94,95]. We found no correlation in body condition between ASY males and mates, but a positive correlation between SY males’ and mates. Therefore, body condition could be affecting mating behavior if low body condition competitively inferior SY males are making the “best of a bad job” by mating to low body condition females [26,93].

The lack of perceivable differences between SY males’ and females’ body coloration is compatible with both hypotheses (female mimicry and status signaling) of the evolution and maintenance of DPM. However, as males’ singing behavior reveals their presence within the breeding site [42], the combined effect of song and dull coloration suggests SY males honestly reveal their sex and status to conspecifics. We had multiple sightings of ASY males in severe and eventually lethal confrontations against each other but never against SY males breeding in the area or neighboring boxes (VM, pers. obs.). This may indicate that singing SY plumaged males simultaneously disclose their sex, age, and subordinate status, reducing the likelihood of receiving severe attacks from ASY males. Regardless of the benefits acquired by SY males, some studies have found that ASY males tolerate SY males as neighbors [96,97] and increase their own reproductive success by siring offspring at those nests [97,98]. Ongoing studies involving age-specific spatial distribution of nests and paternity analysis in both types of males will help to determine if older males are retaining preferred nest boxes or obtaining extra pair paternity benefits from SY males neighboring nests.
